# MeHg Causes Ultrastructural Changes in Mitochondria and Autophagy in the Spinal Cord Cells of Chicken Embryo

**DOI:** 10.1155/2018/8460490

**Published:** 2018-08-28

**Authors:** Fabiana F. Ferreira, Evelise M. Nazari, Yara M. R. Müller

**Affiliations:** ^1^Instituto de Ciências Naturais Humanas e Sociais, UFMT, Avenida Alexandre Ferronato 1200, Setor Industrial, Sinop, MT 78557287, Brazil; ^2^Departamento de Biologia Celular, Embriologia e Genética, Centro de Ciências Biológicas, UFSC, Campus Universitário, Trindade, Florianópolis, SC 88040-900, Brazil

## Abstract

Methylmercury (MeHg) is a known neurodevelopmental toxicant, which causes changes in various structures of the central nervous system (CNS). However, ultrastructural studies of its effects on the developing CNS are still scarce. Here, we investigated the effect of MeHg on the ultrastructure of the cells in spinal cord layers. Chicken embryos at E3 were treated* in ovo* with 0.1 *μ*g MeHg/50 *μ*L saline solution and analyzed at E10. Then, we used transmission electron microscopy (TEM) to identify possible damage caused by MeHg to the structures and organelles of the spinal cord cells. After MeHg treatment, we observed, in the spinal cord mantle layer, a significant number of altered mitochondria with external membrane disruptions, crest disorganization, swelling in the mitochondrial matrix, and vacuole formation between the internal and external mitochondrial membranes. We also observed dilations in the Golgi complex and endoplasmic reticulum cisterns and the appearance of myelin-like cytoplasmic inclusions. We observed no difference in the total mitochondria number between the control and MeHg-treated groups. However, the MeHg-treated embryos showed an increased number of altered mitochondria and a decreased number of mitochondrial fusion profiles. Additionally, unusual mitochondrial shapes were found in MeHg-treated embryos as well as autophagic vacuoles similar to mitophagic profiles. In addition, we observed autophagic vacuoles with amorphous, homogeneous, and electron-dense contents, similar to the autophagy. Our results showed, for the first time, the neurotoxic effect of MeHg on the ultrastructure of the developing spinal cord. Using TEM we demonstrate that changes in the endomembrane system, mitochondrial damage, disturbance in mitochondrial dynamics, and increase in mitophagy were caused by MeHg exposure.

## 1. Introduction

The toxicity of methylmercury (MeHg) is a well-documented phenomenon; its effect on the developing central nervous system (CNS) has been investigated in humans since the 1970s, following environmental accidents [1–5]. Different experimental models, such as rats (*Rattus norvegicus*) [6–8], mice (*Mus musculus*) [9–11], chicks (*Gallus domesticus*) [12, 13], fish (*Danio rerio*) [14], and amphibians (*Xenopus laevis*) [15] have also been used to investigate the cytotoxic effects of this organometal. Even at low concentrations MeHg causes cellular damage in the developing CNS that can lead to permanent impairment [16–21]. The main reason proposed for these severe effects is related to the wide window of susceptibility to exogenous agents presented by the developing CNS [22, 23] which in the initial stage is not protected by the blood-brain barrier [24–26]. Studies of developmental neurotoxicity generally use brain structures (brain, cerebellum, and hippocampus) as an organ model, given the well-documented behavioral (learning and memory deficit) and motor changes caused by MeHg poisoning [16, 17, 21, 27–29]. However, the spinal cord is also an interesting model for neurodevelopmental toxicity studies because its structural organization is less complex than the encephalon and it has fewer tissue layers, allowing observation of the neurotoxic effects on cells at different stages of differentiation [14, 15, 30].

The effects of MeHg can be observed in different stages of neurogenesis; these effects can be observed in neural progenitors at the initial stage [31] which had reduced proliferation after exposure to the metal [15, 20, 30, 32] in later stages; when the neural and glial lineages are being established, MeHg disrupts the cell differentiation process, causing alterations in the expression of genes related to maintaining the characteristics of the neuronal and glial cell lineages [15, 33, 34]. An increase in programmed cell death also occurs in neural cells exposed to MeHg [15, 31, 33, 35], especially in the precursors that appear to be more susceptible than more differentiated cells [21, 31].

Indeed, many studies of neurodevelopmental toxicity indicate a direct relationship between neurogenic disorders and an increase in the production of reactive oxygen species (ROS) [19, 36–41]. Also associated with the increase in ROS production is an imbalance in the antioxidant defense system with changes in glutathione, glutathione peroxidase, and glutathione reductase concentration [11, 38, 40]. Additionally, the imbalance in the antioxidant defense system can increase lipid peroxidation, with damage to membranes and DNA (nuclear and mitochondrial), and impair mitochondrial activity [38, 40, 42].

Mitochondria appear to be an important target of MeHg cytotoxicity and considering that developing nervous tissue makes high energetic demands, damage to the mitochondria may result in risks to cell survival [13, 20, 27, 30].

In addition, mitochondria play an important role in cell death signaling by activating caspases, which appear to be affected by MeHg exposure [20, 31, 39, 43], most often resulting in apoptotic cell death [15, 32, 33, 35, 42]. This type of programmed cell death can be activated simultaneously by several signaling pathways, with or without the participation of mitochondria [20, 43, 44], and shows certain specific characteristics with morphological and biochemical changes, such as cell shrinkage, chromatin condensation, and regulated intracellular degradative processes maintaining plasma membrane integrity [44, 45]. In response to such damage, other types of programmed cell death can be triggered, for example, death by autophagy, previously observed in systems exposed to heavy metals [46–48]. We observed DNA damage associated with cell death in the spinal cord of chicken embryos in an earlier study [30]. Although we did not observe morphological changes, the reduction in the thickness of the spinal cord layers was significant, demonstrating MeHg developing neurotoxicity, even at a very low dose.

Taking into account previous data in CNS chick embryos [13, 30] and the few studies on the effects of MeHg on CNS ultrastructure [4, 49], our objective in this study was to analyze the ultrastructure of the organelles, especially the mitochondria, of the spinal cord cells of chicken embryos exposed to MeHg, to better understand the effects of this organometal on the developing CNS.

## 2. Materials and Methods

### 2.1. MeHg Treatment* In Ovo*

Fertilized eggs of* G. domesticus* were obtained from a commercial hatchery (Tyson Foods Brazil Ltd, Brazil). The eggs were weighed (66.6 ± 4.7 g) and transferred to an incubator at 38.0°C and 65.0% humidity. Prenatal acute MeHg exposure was performed at embryonic day 3 (E3) [50]. The embryos received a single dose of 0.1 *μ*g of Methylmercury II chloride (Sigma-Aldrich, USA) diluted in 50 *μ*L of saline solution, administered into the yolk sac near the vitellin vessels. Untreated control embryos received only 50 *μ*L of saline solution (NaCl 0.9%). The dose of MeHg used in this study was determined according to Heinz et al. [51, 52] and on the basis of a previous study performed by our group [13, 30]. After treatment, each egg was returned to the incubator and embryos were monitored daily* in ovo* up to embryonic day 10 (E10). At E10, the embryos were anesthetized by cooling to 4°C for 15-20 min, removed from the eggshell, and washed in saline solution. After morphological and morphometric analysis of the embryos, spinal cord was dissected and submitted to the procedures. The experiments were carried out according to the Ethics Committee for Animal Research of the Universidade Federal de Santa Catarina (UFSC), Florianópolis, Brazil (approval n°. 355/CEUA /UFSC).

### 2.2. Transmission Electron Microscopy (TEM)

For observation under the transmission electron microscope (TEM), spinal cord fragments were fixed in 2.5% glutaraldehyde and 4.0% paraformaldehyde diluted in 0.1 M sodium cacodylate buffer for 24 h at 4°C and postfixed with 1% osmium tetroxide for 2 h. Dehydration was performed in an acetone gradient series and the samples were embedded in Spurr's resin. Semithin sections (700 nm) were analyzed with 1% toluidine blue and thin spinal cord sections (60-90 nm) were stained with aqueous 5% uranyl acetate, followed by 1% lead citrate. Material analysis and image capture were performed in a JEOL JEM-1011 transmission electron microscope (operating at 80 kV).

### 2.3. Data Analysis

For ultrastructural comparative analysis and mitochondria quantification by TEM, 5 embryos (3 sections per embryo, 5 random fields per section) were analyzed at 20,000x magnification, in a total of 50 ultramicrographs per group. Quantitative mitochondrial analysis was performed according to Glaser et al. [49]. ImageJ software (NIH) was used and the ratio of mitochondria number/*μ*m^2^ in spinal cord was established. Quantitative data were analyzed using Statistica® 10.0 for Windows. MeHg-treated and untreated control embryos were analyzed with Student's unpaired t-test. All data were expressed as mean ± SEM, and P < 0.05 was considered statistically significant.

## 3. Results

### 3.1. General Remarks

**Figure 1:**
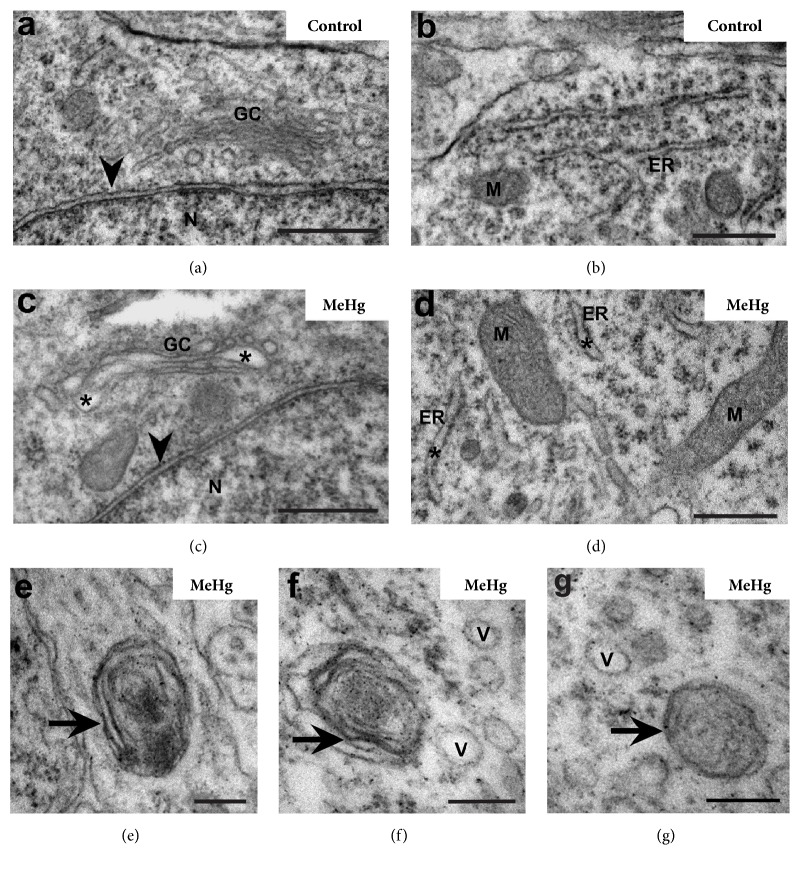
*Effect of MeHg on the endomembrane system of the spinal cord embryos*. Electron micrograph of the E10 spinal cord mantle layer showing Golgi complex (GC) and endoplasmic reticulum (ER) in control and MeHg-treated embryos. Slightly dilated cisterns (*∗*) in the GC and ER and myelin-like membranous inclusions (arrows) were observed. Note the vesicles (V) close to the myelin-like inclusions. Nuclear envelope (arrowhead), nucleus (N), and mitochondria (M). Scale bars: (a–d) 0.5 *μ*m; (e–g) 200 nm.

**Figure 2:**
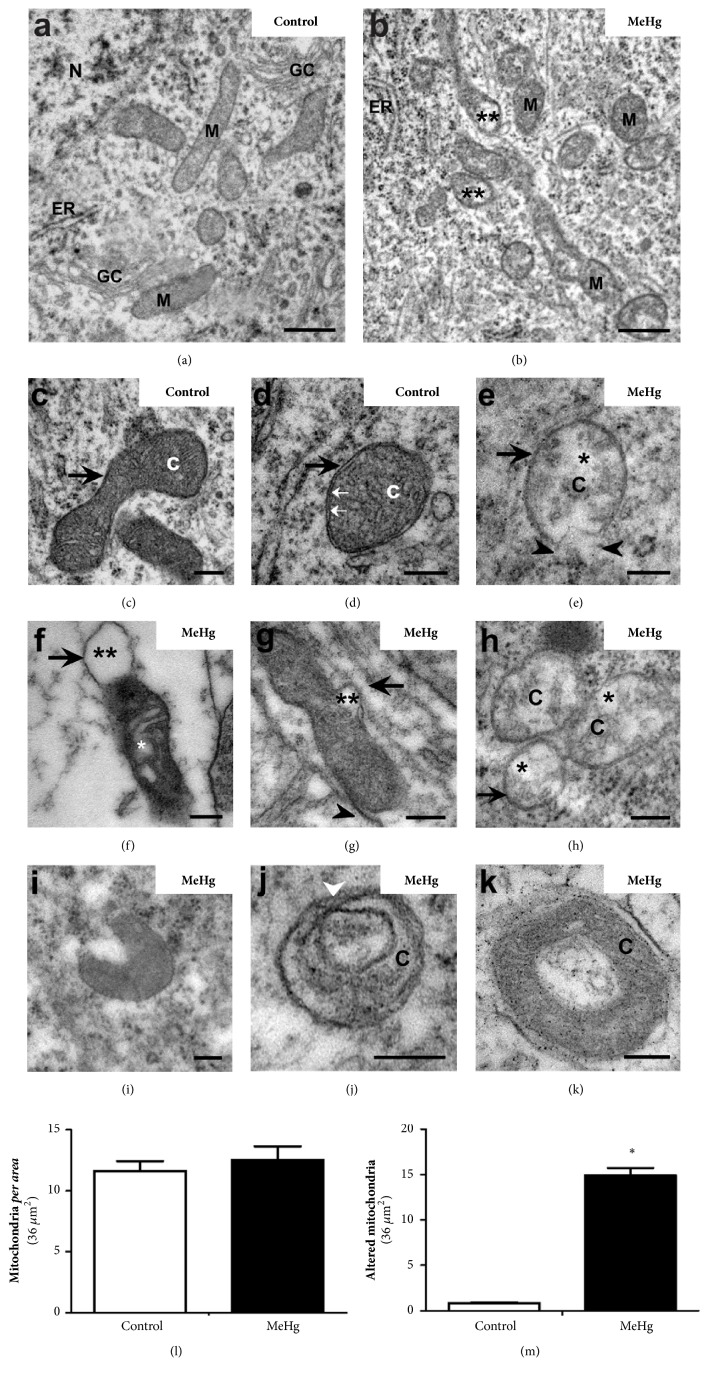
*Effects of MeHg on mitochondrial structure in spinal cord of E10 embryos*. Electron micrograph showing mitochondria in the mantle layer of control and MeHg-treated embryos. Mitochondria (M) in control embryos showed mitochondrial crests (C), internal mitochondrial membrane (IMM, white arrow), and external mitochondrial membrane (EMM, black arrow) visualized in longitudinal (a, c) and transverse planes (a, d). Mitochondria of the MeHg-treated embryos showed ruptures in the EMM (black arrowhead), loss and disorganization of crests, swelling (*∗*) in the mitochondrial matrix, and vacuolization (*∗∗*) between IMM and EMM. Unusual mitochondrial shapes, cup-like (i) and donut-like (j-k), were observed in MeHg-treated embryos. Fusion (white arrowhead) in mitochondrial donut-like shape. The graphs show the total number of mitochondria (l) and the number of altered mitochondria (m) in control and MeHg-treated groups. *∗* indicates P < 0.05. Nucleus (N), endoplasmic reticulum (ER), and Golgi complex (GC). Scale bars: (a-b) 1 *μ*m; (c–k) 200 nm.

In a previous study our group showed that a single injection of 0.1 *μ*g MeHg/50 *μ*L of saline solution in E3 chicken embryos caused a reduction in the thickness of spinal cord layers but did not cause morphological alterations in cytoarchitecture at E10 [30]. In this study, where we analyzed the E10 spinal cord using a TEM, we observed changes in some membranous organelles of the mantle layer cells in MeHg-treated embryos. These embryos also displayed alterations in the endoplasmic reticulum (ER), Golgi complex (GC), and mitochondria (Figures 1 and 2). Also, myelin-like cytoplasmic inclusions, figures similar to mitophagy, and large vacuoles similar to autophagic vacuoles, indicating intense autophagy, were observed.

### 3.2. Effect of MeHg on Cell Endomembranes

The spinal cord cells in MeHg-treated embryos showed GC and ER (Figures 1(a)-1(d)) with slightly dilated cisterns and fewer ribosomes adhering to the ER membrane. Myelin like concentric membranous inclusions (Figures 1(e)-1(g)) and small vesicles (Figures 1(f) and 1(g)) were also observed in embryos exposed to MeHg. The nuclear membrane showed no differences between the MeHg-treated and control embryos (Figures 1(a) and 1(c)).

### 3.3. Mercury Effect on Mitochondrial Structure

Cells of the control embryos displayed elongated tubular mitochondria with many crests in the internal mitochondrial membrane (IMM) (Figures 2(a), 2(c), and 2(d)). The mitochondria observed in MeHg-treated embryos maintained the tubular morphology but exhibited loss of mitochondrial crests and swelling in the mitochondrial matrix (Figures 2(b) and 2(e)–2(h)). Some mitochondria exhibited disruption in the external mitochondrial membrane (EMM) (Figure 2(e)) and large vacuolization (similar to bubbles) in the space between IMM and EMM (Figures 2(f) and 2(g)). In addition to the severe mitochondrial damage observed, unusual mitochondria shapes were also identified in the MeHg-treated embryos. Mitochondrial forms rarely found in control embryos, such as cup-like shapes (Figure 2(i)) and ring or donut-like shapes (Figures 2(j) and 2(k)), were frequently found in the mantle layer of MeHg-exposed embryos. Although the total number of mitochondria did not differ between control and MeHg-treated embryos, the number of damaged mitochondria was significantly higher in spinal cord cells of the MeHg-treated embryos (P < 0.05) (Figures 2(l) and 2(m)).

### 3.4. Fusion and Fission Mitochondrial Profiles

**Figure 3:**
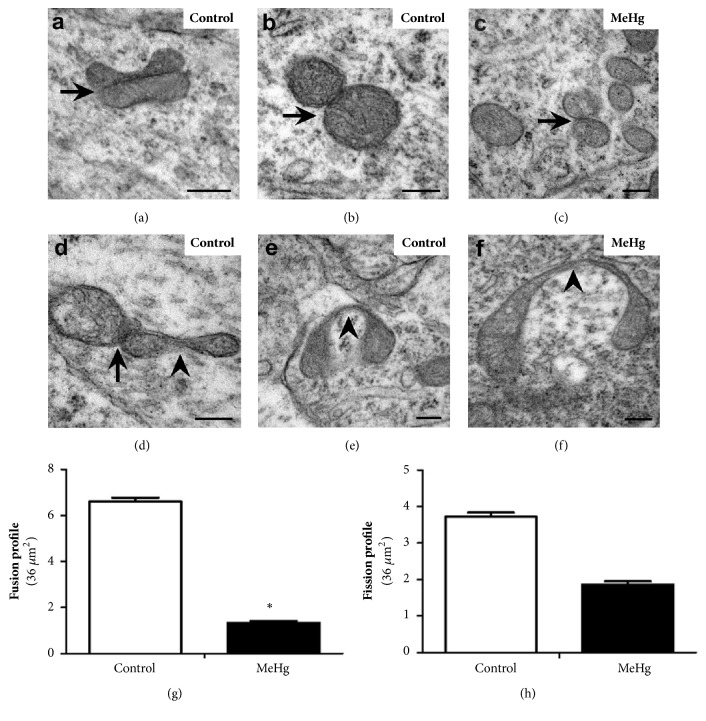
*Effect of MeHg on mitochondrial fusion and fission profiles in spinal cord cells of E10 embryos*. Electron micrograph of the mantle layer in control and MeHg-treated embryos. The graphs show the number of the fusion (g) and fission (h) mitochondrial profiles in both analyzed groups. Mitochondrial fusion (black arrow) and fission (arrowhead). *∗*Indicates P < 0.05. Scale bars: (a–f) 200 *η*m.

The fusion and fission mitochondrial profiles were observed in control (Figures 3(a), 3(b), 3(d), and 3(e)) and MeHg-treated embryos (Figures 3(c) and 3(f)). A decrease in mitochondrial fusion was observed in MeHg-treated embryos (Figure 3(g)). However, mitochondrial fission was not affected by the MeHg dose used here; we did not observe a difference between the control and MeHg-treated embryos (Figure 3(h)).

### 3.5. Autophagy in MeHg-Treated Embryos

**Figure 4:**
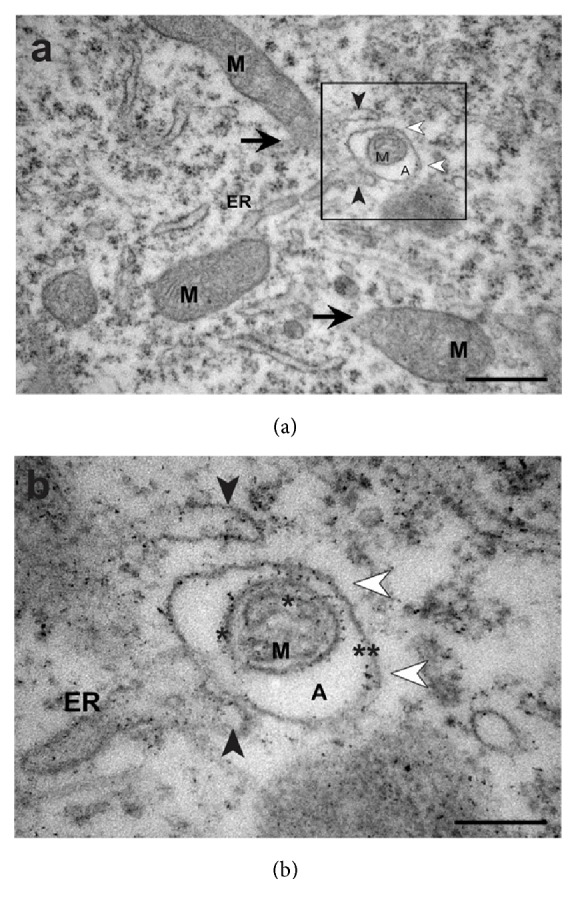
*Effect of MeHg on vacuole formation in spinal cord cells of E10 embryos*. Electron micrograph of the mantle layer showing the mitophagic profile in the black box (a). The insert in (b) shows a detail of mitochondria (M) surrounded by the autophagic vacuole membrane (white arrowhead). In detail (b) internal mitochondrial membranes (asterisk) and external mitochondrial membranes (double asterisk) are visible. Endoplasmic reticulum (ER) can be seen close to the autophagic vacuole (A) maintaining contact with it by membrane extensions (black arrowhead). Scale bars: (a) 500 *μ*m; (b) 200 *η*m.

Autophagic bodies or vacuoles containing mitochondria were observed in MeHg-treated embryos (Figures 4(a) and 4(b)). Additionally, the presence of mitochondrial membranes (IMM and EMM) and disruption in EMM were noted. The damaged mitochondria were surrounded by an outer membrane, which maintained contact with the endoplasmic reticulum membrane (Figure 4(b)).

**Figure 5:**
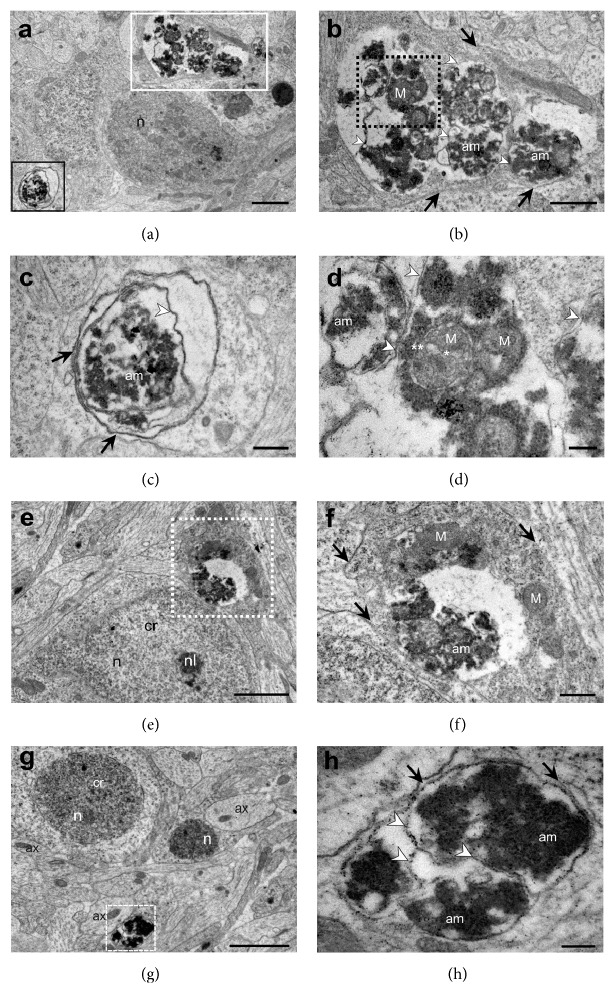
*Autophagic vacuoles in spinal cord of MeHg-treated embryos*. Cells in mantle layer show autophagic vacuoles in different stages of autolysis (a–h). Plasma membranes delimit the cells (black arrow) and inner membranes delimit compartments similar to autophagic vacuoles (white arrowhead). Mitochondria (M) with IMM (*∗*) and EMM (*∗∗*) still preserved, inside early autophagic vacuole (a-b, d). Later autophagic vacuoles with amorphous (am), homogeneous, and more electron-dense content (a, c, g, h). (a) Spinal cord cells in lower magnification. (b) Magnification of the cell inside of the white box in (a). (c) Magnification of the cell inside the black box in (a). (d) Magnification of the autophagic vacuoles of the black dotted box in (b). (e) Spinal cord mantle layer cells. In the white dotted box, there is a cell in autophagy. (f) Magnification of the cell in the death process inside the white dotted box in (e). (g) Cell with amorphous and more electron dense cytoplasm in white dashed box. (h) Magnification of the highlighted cell in (g). Axon (ax) of neuronal cell in transverse section, chromatin (cr), nucleus (N), and nucleolus (nl). Scale bars: (a, e) 2 *μ*m; (b) 1.0 *μ*m; (c) 0.5 *μ*m; (d,f) 0.2 *μ*m.

Many cells containing autophagic vacuoles were found in the mantle layer of the MeHg-treated embryos (Figures 5(a)–5(f)). Mitochondria in the process of autolysis were found in some vacuoles (Figures 5(b) and 5(d)); it is possible to identify the mitochondria delimited by vacuolar membranes, similar to mitophagy (Figure 5(d)). In other vacuoles, the content was amorphous and electron dense, so it was not possible to identify any structure (Figures 5(b)–5(f)). Some cells appeared to be in an advanced degree of autophagy (Figures 5(c), 5(e), and 5(f)). These cells only have amorphous content inside the vacuoles, which appear to converge to a single and large autophagic vacuole (Figures 5(c) and 5(f)). No cells containing autophagic vacuoles in MeHg-treated embryos displayed plasma membrane disruption or cytoplasmic extrusion.

## 4. Discussion

Our data showed that a single dose of MeHg caused significant ultrastructural changes in the endomembrane system and mitochondria of the spinal cord in chicken embryos. This neurodevelopmental toxicity study demonstrates that the mitochondria are an important target of MeHg. Treated embryos showed a higher number of altered mitochondria (with disruption, swelling, or vacuolization) and unusual mitochondrial cup- and donut-like shapes compared with control embryos. Previous work using light microscopy analysis has demonstrated the harmful effects of the same dose of MeHg [30], where it caused reduction in the thickness of the ependymal, mantle, and marginal layers. The effects were mainly observed in the mantle layer, where DNA damage, decrease in proliferation, and increase in cell death were observed. Other works, with the same dose of MeHg used here, showed a reduction in the number of Purkinje cells [13] which are associated with neuromotor and learning deficits.

Studies performed in different models of neurodevelopmental toxicity showed that MeHg causes an increase in ROS, leading to oxidative stress and disturbance in the antioxidant defense system, enhancing MeHg neurotoxicity [11, 13, 36, 40, 41, 53]. Lipid peroxidation has been reported to be a consequence of MeHg toxicity [38–40, 42, 53, 54] and is related to hydrogen peroxide and its precursor superoxide anion causing the formation of hydroxyl radicals that remove hydrogen atoms from the lipid chains of cell membranes [10, 55]. Another important factor that appears to contribute to the appearance of oxidation signals is the large amount of polyunsaturated fatty acids present in the CNS; these are more sensitive to peroxidation [56]. In addition to the lipids present in the plasma membrane, some cellular organelles such as lysosomes [57] and mitochondrial membranes also represent important peroxidation targets [39].

Dilation of ER cisternae and mitochondrial changes have been observed in nonneural tissues exposed to MeHg* in vivo *[58] and changes in mitochondria and membranous cytoplasmic inclusions were observed in renal cells exposed* in vitro* to mercury chloride (HgCl) [45]. Together, these data suggest that MeHg might alter the lipid metabolism in the developing spinal cord, causing ruptures and changes in mitochondria, dilations of the cisterns of the ER and GC, and the appearance of myelin-like cytoplasmic inclusions. MeHg oxidative action targets mitochondria organelles; this causes damage to the mitochondria's structures and compromises the activity of proteins essential for its function, as well as increasing ROS generation [40]. Some of the damage observed in the mitochondrial structure in spinal cord embryos may have been caused by lipid peroxidation, but some ion channels may also have been altered, leading to swelling and the vacuolation observed in the mitochondria in our study and in others which have used mercury as a cytotoxic agent [45, 49, 58]. Among the few studies that have analyzed the ultrastructural effects of mercury on the nervous system, Glaser et al. [49] demonstrated that MeHg causes changes in the mitochondrial crests in the cerebral cortex of adult rats. Even in nonneural tissues, such as the kidneys and muscles, the effects of mercury on mitochondria appear to be stereotypical, showing the same characteristics we observed, such as swelling in the matrix, crest disorganization, and vacuolization between the internal and external mitochondrial membranes [45, 58, 59].

An unexpected result of our study was the identification of unusual cup- and donut-like mitochondrial shapes in the MeHg-treated embryos. The mitochondria in the normal state may present a network morphology of tubules, curved or cup-shape and ring or donut-shape, which are the result of autofusion. Mitochondria are very dynamic organelles that can alter their shape, to fuse or to divide in conditions of high energy demand or stress. These strategies serve to protect their structures and to optimize their activity in an attempt to restore cellular homeostasis [60, 61]. Changes in mitochondrial shape are important indicators of cell stress [62–65] so the appearance of cup and donut-like mitochondrial forms in the spinal cord cells of MeHg-treated embryos seem to indicate disturbance in cellular homeostasis, since these mitochondrial forms often appear under oxidative stress [65–70].

In the present study, a higher number of altered mitochondria were observed in MeHg-treated embryos, suggesting that this organometal may cause serious damage to mitochondrial structure and function in the developing spinal cord. Mitochondria have a functional versatility that is accompanied by morphological complexity [71] and repetitive cycles of fusion and fission are fundamental to the mitochondrial dynamics. Fission and fusion dynamics establish the size, number, and shape of the mitochondria and allow the mixing of the mitochondrial contents, including proteins, lipids, and DNA. Neurons, due to their high energy demand, contain many mitochondria, which are highly active in movement and fission and fusion dynamics [72].

Glaser et al. [49] analyzed the mitochondria quantitatively and evaluated their size, identifying a higher number and larger size of the mitochondria in the cerebellar cortex, suggesting that MeHg may have altered the fission and fusion mitochondrial dynamics in the nervous system. Fission and fusion mitochondrial dynamics are controlled by groups of dynamins: GTPases [60, 73] among them standout DRP-1 (dynamin-related protein 1), Mfn (Mitofusin), and OPA (optic atrophy protein-1) [74]. Mitochondrial fusion is particularly important in the nervous system, helping the neurons to meet the high demand for ATP for neuronal function and maintaining an adequate level of bioenergy capacity [70, 75, 76].

Mitochondria continuously produce superoxide anions, which are highly reactive, as a subproduct of electron transport. ROS causes damage to proteins, lipids, and mitochondrial DNA, so mitochondria have proteases to eliminate damaged structures. This repair system works as mitochondrial quality control, detecting and correcting minor damage, without the need to change the fission or fusion rate [77]. Another level of quality control involves the elimination of mitochondria by autophagy, a process which is necessary to maintain a healthy mitochondrial network [78, 79]. The contribution of ER to autophagy vacuole formation is evident in our analysis. Many studies have demonstrated ER participation in vacuole formation and the interchange of molecules with mitochondria for the maintenance of cellular homeostasis [70, 80]. Mitophagy may occur associated with mitochondrial fission, by separating the functional mitochondria from damaged portions, directing the latter to mitophagy. In cases of slight damage to the mitochondria, fusion may minimize deleterious effects by increasing the mitochondrial area in an attempt to neutralize the damage; in case of more severe damage, the mitochondria are selected for mitophagy [79].

Data obtained on spinal cord MeHg-treated embryos showed a significant reduction in the number of mitochondrial fusion profiles. Our results are similar to those observed by Lionetti et al. [69] in hepatocytes submitted to oxidative stress* in vitro*, where, in addition to increased ROS formation, decreased expression of Mfn2 and OPA-1 proteins was observed, as well as a reduction in mitochondrial fusion profiles. Another fusion reduction pathway is provided through ubiquitination, membrane extraction, and degradation of outer mitochondrial membrane fusion proteins Mfn1 and Mfn2, via proteasomes [80, 81].

The reduction in fusion profile associated with the increased mitophagy observed in the present study may indicate a combination of protective strategies in the embryonic cells of the spinal cord exposed to MeHg, attempting to eliminate damaged mitochondria. However, since mitochondrial fusion is an important mechanism for neural tissues, due to high energy demand, the disturbance of this mitochondrial dynamic may impair spinal cord development.

Studies carried out in various structures of the CNS show apoptosis cell death caused by MeHg resulting in alterations to the cytoarchitecture of the central nervous system [7, 15, 20, 42].* In vivo* and* in vitro* studies have indicated that MeHg may increase apoptotic cell death in the developing central nervous system, with or without the participation of mitochondrial signaling pathways [15, 30, 31, 33, 42]. Autophagic cell death has been observed in neural and nonneural cells treated with heavy metals, such as cadmium [46, 47], mercury [48], and arsenic [82, 83] at low doses (less than 10 *μ*M). In our study of MeHg-treated embryos, we observed cells with internal compartments delimited by membranes similar to autophagic vacuoles or autophagosomes. These cells were delimited by the plasma membrane, without extrusion of its content, volume increase, or disruption in membrane, indicating that this is not apoptotic or necrotic death [84, 85].

## 5. Conclusions

Our study brings new insights into the effect of MeHg on the ultrastructure of developing neural cells. Here, we have shown that a single dose of MeHg, administered* in ovo*, can disrupt a system in development, causing damage to the mitochondrial ultrastructure, which can, in turn, lead to autophagy.

## Data Availability

The transmission electron microscopy, light microscopy, and mitochondria quantification data used to support this study were deposited in the following repositories: https://repositorio.ufsc.br/handle/123456789/169627,  http://lrda.ccb.ufsc.br/teses-e-dissertacoes/, and  http://catalogodeteses.capes.gov.br/catalogo-teses/ Ferreira, Fabiana de Fatima. Morphological and morphometrical analyses and immunostaining (related to cell proliferation, cell cycle, and cell death) in addition to quantitative analyses of the antioxidant defense system molecules (glutathione, glutathione peroxidase, and glutathione reductase) reported in previous articles of our group were used to support this study and are available at http://dx.doi.org/10.1155/2015/532691 and https://doi.org/10.1093/toxsci/kfn158. These previous studies are cited in relevant places in the text as [13, 30]. Additional information about the present study can be obtained from Fabiana de Fatima Ferreira by e-mail: ffferreira@hotmail.com.
